# *N*-(2-Fluoro­phen­yl)cinnamamide

**DOI:** 10.1107/S1600536810003867

**Published:** 2010-02-06

**Authors:** Aamer Saeed, Rasheed Ahmad Khera, Jim Simpson

**Affiliations:** a Department of Chemistry, Quaid-i-Azam University, Islamabad 45320, Pakistan; b Department of Chemistry, University of Otago, PO Box 56, Dunedin, New Zealand

## Abstract

The title compound, C_15_H_12_FNO, was prepared by the reaction of cinnamoyl chloride with 4-fluoro­aniline and crystallizes with two mol­ecules *A* and *B* in the asymmetric unit. The two unique mol­ecules are closely similar and overlay with an r.m.s. deviation of 0.0819 Å. The fluoro­benzene and phenyl rings are inclined to one another at 73.89 (7) and 79.46 (7)°, respectively, in mol­ecules *A* and *B*. The amide C—N—C(O)—C portions of the mol­ecules are planar (r.m.s. deviations = 0.035 and 0.028 Å) and are inclined at 45.51 (9) and 47.71 (9), respectively, to the fluoro­benzene rings in mol­ecules *A* and *B*. The 2-fluoro­acetamide units and the benzene rings each adopt *E* configurations with respect to the C=C bonds. In the crystal structure, inter­molecular N—H⋯O hydrogen bonds augmented by weak C—H⋯π inter­actions link mol­ecules into rows in a head-to-tail fashion along *a*. Additional weak C—H⋯O contacts further stabilize the packing, forming a three-dimensional network stacked down *a*.

## Related literature

For related structures see: Leiserowitz & Tuval (1978 ▶); Nilofar Nissa *et al.* (2002 ▶, 2004 ▶); Jones & Dix (2008 ▶); Saeed *et al.* (2009 ▶). For details of the Cambridge Structural Database: see Allen (2002 ▶).
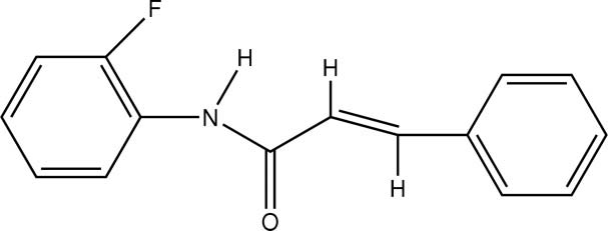


## Experimental

### 

#### Crystal data

C_15_H_12_FNO
*M* *_r_* = 241.26
Monoclinic, 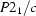

*a* = 9.6634 (12) Å
*b* = 13.0838 (17) Å
*c* = 19.404 (3) Å
β = 99.297 (7)°
*V* = 2421.0 (5) Å^3^
*Z* = 8
Mo *K*α radiation
μ = 0.09 mm^−1^
*T* = 89 K
0.64 × 0.30 × 0.16 mm

#### Data collection

Bruker APEXII CCD diffractometer
Absorption correction: multi-scan (*SADABS*; Bruker, 2006 ▶) *T*_min_ = 0.696, *T*_max_ = 1.000
24964 measured reflections
4376 independent reflections
3312 reflections with *I* > 2σ(*I*)
*R*_int_ = 0.068

#### Refinement

*R*[*F* ^2^ > 2σ(*F* ^2^)] = 0.050
*wR*(*F* ^2^) = 0.156
*S* = 1.07
4376 reflections
331 parameters
H atoms treated by a mixture of independent and constrained refinement
Δρ_max_ = 0.30 e Å^−3^
Δρ_min_ = −0.36 e Å^−3^

### 

Data collection: *APEX2* (Bruker 2006 ▶); cell refinement: *APEX2* and *SAINT* (Bruker 2006 ▶); data reduction: *SAINT*; program(s) used to solve structure: *SHELXS97* (Sheldrick, 2008 ▶); program(s) used to refine structure: *SHELXL97* (Sheldrick, 2008 ▶) and *TITAN2000* (Hunter & Simpson, 1999 ▶); molecular graphics: *SHELXTL* (Sheldrick, 2008 ▶) and *Mercury* (Macrae *et al.*, 2008 ▶); software used to prepare material for publication: *SHELXL97*, *enCIFer* (Allen *et al.*, 2004 ▶), *PLATON* (Spek, 2009 ▶) and *publCIF* (Westrip, 2010 ▶).

## Supplementary Material

Crystal structure: contains datablocks global, I. DOI: 10.1107/S1600536810003867/hg2641sup1.cif

Structure factors: contains datablocks I. DOI: 10.1107/S1600536810003867/hg2641Isup2.hkl

Additional supplementary materials:  crystallographic information; 3D view; checkCIF report

## Figures and Tables

**Table 1:** Hydrogen-bond geometry (Å, °) *CgA* and *CgB* are the centroids of the fluoro­benzene rings in mol­ecules *A* and *B* respectively.

*D*—H⋯*A*	*D*—H	H⋯*A*	*D*⋯*A*	*D*—H⋯*A*
N1*A*—H1*NA*⋯O1*B*	0.88 (3)	1.98 (3)	2.851 (2)	173 (2)
N1*B*—H1*NB*⋯O1*A*^i^	0.81 (3)	2.07 (3)	2.870 (2)	170 (2)
C14*A*—H14*A*⋯O1*A*^ii^	0.95	2.50	3.410 (3)	160
C14*B*—H14*B*⋯O1*B*^iii^	0.95	2.59	3.476 (3)	155
C9*B*—H9*B*⋯*CgA*^iv^	0.95	2.89	3.679 (2)	141
C5*A*—H5*A*⋯*CgB*^v^	0.95	2.80	3.621 (2)	149

Symmetry codes: (i) 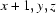; (ii) 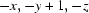; (iii) 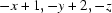; (iv) 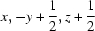; (v) 
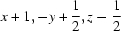
.

**Fig. 1.**
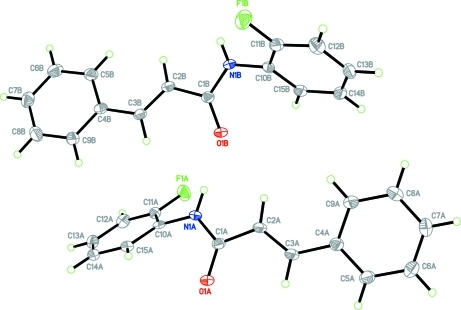
The asymmetric unit of (I) with displacement ellipsoids for the non-hydrogen atoms drawn at the 50% probability level.

**Fig. 2.**
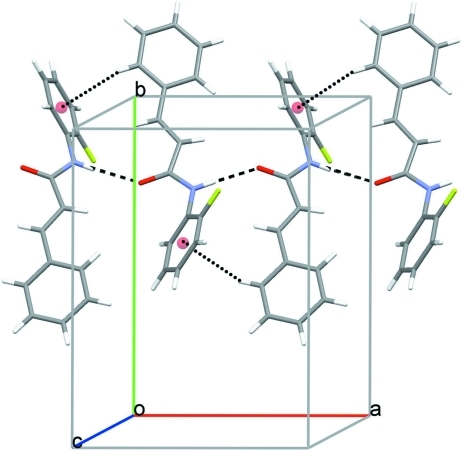
N—H···O hydrogen bonds (dashed lines) and weak C–H···π interactions (dotted lines) forming chains down b.

**Fig. 3.**
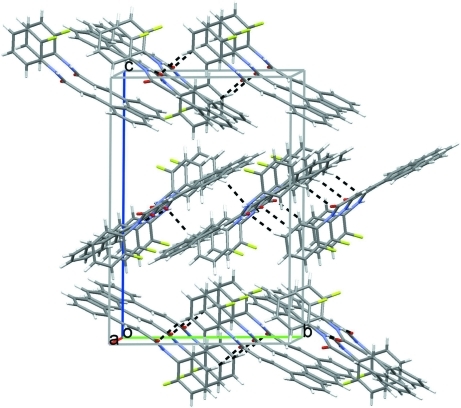
Crystal packing for (I) viewed down the a axis with hydrogen bonds drawn as dashed lines.

## supplementary crystallographic information

### Comment

The background to this work has been described in a previous paper (Saeed *et al.* 2009). The title compound, C_15_H_12_FNO (I), was prepared by the reaction of cinnamoyl chloride with 4-fluoroaniline. The compound crystallises with two molecules A and B in the asymmetric unit of the monoclinic unit cell. These two unique molecules are closely similar and overlay with an rms deviation of 0.0819Å (Macrae *et al.*, 2008). The fluorobenzene and benzene rings are inclined at 73.89 (7)° and 79.46 (7)° respectively in the two molecules. The amide C10–N1–C1(O1)–C2 portions of the molecules are planar (rms deviations 0.035 and 0.028 Å) and are inclined at 45.51 (9)° and 47.71 (9) respectively to the fluorobenzene rings. The 2-fluoroacetamide units and the benzene rings each adopt *E* configurations with respect to the C═C bonds. A search of the Cambridge Database (Allen, 2002) reveals only one closely related halobenzene derivative (Nilofar Nissa *et al.*, 2004) but the structures of a number of other cinnamanilide compounds are known (Leiserowitz & Tuval 1978; Nilofar Nissa *et al.*, 2002; Jones & Dix, 2008; Saeed *et al.*, 2009).

In the crystal structure, intermolecular N1A—H1NA···O1B and N1B—H1NB···O1A hydrogen bonds, augmented by weak C–H···π interactions involving the two fluorobenzene rings, link molecules into rows along *a*, Fig. 2. Additional weak C—H···O contacts further stabilise the packing, forming a three-dimensional network stacked down *a*, Table 1 & Fig. 3.

### Experimental

Cinnamoyl chloride (5.4 mmol) in CHCl_3_ was treated with 4-fluoroaniline (21.6 mmol) under a nitrogen atmosphere at reflux for 2 h. Upon cooling, the reaction mixture was diluted with CHCl_3_ and washed consecutively with aqueous 1 *M* HCl and saturated aq NaHCO_3_. The organic layer was dried over anhydrous magnesium sulfate and concentrated under reduced pressure. Crystallization of the residue from CHCl_3_ afforded the title compound (87%) as colourless needles: Anal. calcd. for C_15_H_12_FNO,: C, 74.67; H, 5.01; N, 5.81%; found: C, 74.69; H, 5.16; N, 5.94%.

### Refinement

The H atoms bound to N1A and N1B were located in a difference map and refined isotropically. All other H-atoms were positioned geometrically and refined using a riding model with d(C—H) = 0.95 Å, U_iso_ = 1.2U_eq_ (C).

### Crystal data

**Table:**

C_15_H_12_FNO	*F*(000) = 1008
*M**_r_* = 241.26	*D*_x_ = 1.324 Mg m^−^^3^
Monoclinic, *P*2_1_/*c*	Mo *K*α radiation, λ = 0.71073 Å
Hall symbol: -P 2ybc	Cell parameters from 5945 reflections
*a* = 9.6634 (12) Å	θ = 2.6–25.1°
*b* = 13.0838 (17) Å	µ = 0.09 mm^−^^1^
*c* = 19.404 (3) Å	*T* = 89 K
β = 99.297 (7)°	Block, pale yellow
*V* = 2421.0 (5) Å^3^	0.64 × 0.30 × 0.16 mm
*Z* = 8	

### Data collection

**Table:**

Bruker APEXII CCD diffractometer	4376 independent reflections
Radiation source: fine-focus sealed tube	3312 reflections with *I* > 2σ(*I*)
graphite	*R*_int_ = 0.068
ω scans	θ_max_ = 25.3°, θ_min_ = 1.9°
Absorption correction: multi-scan (*SADABS*; Bruker, 2006)	*h* = −11→11
*T*_min_ = 0.696, *T*_max_ = 1.000	*k* = −15→15
24964 measured reflections	*l* = −23→23

### Refinement

**Table:**

Refinement on *F*^2^	Primary atom site location: structure-invariant direct methods
Least-squares matrix: full	Secondary atom site location: difference Fourier map
*R*[*F*^2^ > 2σ(*F*^2^)] = 0.050	Hydrogen site location: inferred from neighbouring sites
*wR*(*F*^2^) = 0.156	H atoms treated by a mixture of independent and constrained refinement
*S* = 1.07	*w* = 1/[σ^2^(*F*_o_^2^) + (0.0869*P*)^2^ + 1.0345*P*] where *P* = (*F*_o_^2^ + 2*F*_c_^2^)/3
4376 reflections	(Δ/σ)_max_ < 0.001
331 parameters	Δρ_max_ = 0.30 e Å^−^^3^
0 restraints	Δρ_min_ = −0.36 e Å^−^^3^

### Special details

**Table:**

Geometry. All esds (except the esd in the dihedral angle between two l.s. planes) are estimated using the full covariance matrix. The cell esds are taken into account individually in the estimation of esds in distances, angles and torsion angles; correlations between esds in cell parameters are only used when they are defined by crystal symmetry. An approximate (isotropic) treatment of cell esds is used for estimating esds involving l.s. planes.
Refinement. Refinement of *F*^2^ against ALL reflections. The weighted *R*-factor wR and goodness of fit *S* are based on *F*^2^, conventional *R*-factors *R* are based on *F*, with *F* set to zero for negative *F*^2^. The threshold expression of *F*^2^ > σ(*F*^2^) is used only for calculating *R*-factors(gt) etc. and is not relevant to the choice of reflections for refinement. *R*-factors based on *F*^2^ are statistically about twice as large as those based on *F*, and *R*- factors based on ALL data will be even larger.

### Fractional atomic coordinates and isotropic or equivalent isotropic displacement parameters (Å^2^)

**Table:**

	*x*	*y*	*z*	*U*_iso_*/*U*_eq_	
N1A	0.2567 (2)	0.71300 (15)	0.02767 (10)	0.0190 (4)	
H1NA	0.342 (3)	0.735 (2)	0.0245 (12)	0.023*	
C1A	0.1444 (2)	0.75857 (17)	−0.01166 (11)	0.0177 (5)	
O1A	0.02319 (15)	0.72730 (12)	−0.01255 (8)	0.0220 (4)	
C2A	0.1794 (2)	0.85024 (17)	−0.04971 (11)	0.0181 (5)	
H2A	0.2725	0.8596	−0.0585	0.022*	
C3A	0.0817 (2)	0.92016 (18)	−0.07189 (11)	0.0199 (5)	
H3A	−0.0105	0.9048	−0.0639	0.024*	
C4A	0.0985 (2)	1.01740 (18)	−0.10691 (11)	0.0191 (5)	
C5A	−0.0136 (2)	1.08608 (18)	−0.11720 (12)	0.0229 (5)	
H5A	−0.0991	1.0685	−0.1019	0.027*	
C6A	−0.0017 (3)	1.18006 (19)	−0.14953 (12)	0.0251 (6)	
H6A	−0.0792	1.2257	−0.1564	0.030*	
C7A	0.1223 (3)	1.20718 (19)	−0.17169 (12)	0.0245 (5)	
H7A	0.1303	1.2712	−0.1938	0.029*	
C8A	0.2356 (2)	1.13992 (19)	−0.16129 (11)	0.0235 (5)	
H8A	0.3214	1.1586	−0.1759	0.028*	
C9A	0.2240 (2)	1.04614 (18)	−0.12984 (11)	0.0212 (5)	
H9A	0.3015	1.0006	−0.1236	0.025*	
C10A	0.2461 (2)	0.63122 (17)	0.07449 (12)	0.0179 (5)	
C11A	0.3173 (2)	0.63675 (17)	0.14283 (12)	0.0201 (5)	
F1A	0.39762 (14)	0.72109 (10)	0.16188 (7)	0.0294 (4)	
C12A	0.3107 (2)	0.56122 (19)	0.19135 (12)	0.0244 (5)	
H12A	0.3604	0.5676	0.2376	0.029*	
C13A	0.2297 (2)	0.47516 (19)	0.17133 (12)	0.0241 (5)	
H13A	0.2231	0.4222	0.2041	0.029*	
C14A	0.1585 (2)	0.46671 (18)	0.10335 (12)	0.0216 (5)	
H14A	0.1041	0.4075	0.0896	0.026*	
C15A	0.1665 (2)	0.54433 (17)	0.05545 (12)	0.0194 (5)	
H15A	0.1169	0.5380	0.0092	0.023*	
N1B	0.7349 (2)	0.79010 (15)	−0.03553 (10)	0.0187 (4)	
H1NB	0.814 (3)	0.769 (2)	−0.0341 (13)	0.022*	
C1B	0.6485 (2)	0.74513 (17)	0.00412 (11)	0.0170 (5)	
O1B	0.52817 (15)	0.77646 (12)	0.00522 (8)	0.0216 (4)	
C2B	0.7098 (2)	0.65447 (17)	0.04371 (11)	0.0177 (5)	
H2B	0.8086	0.6452	0.0519	0.021*	
C3B	0.6277 (2)	0.58575 (17)	0.06798 (11)	0.0184 (5)	
H3B	0.5299	0.5994	0.0594	0.022*	
C4B	0.6714 (2)	0.49131 (18)	0.10655 (11)	0.0182 (5)	
C5B	0.8115 (2)	0.47122 (19)	0.13481 (12)	0.0232 (5)	
H5B	0.8819	0.5198	0.1293	0.028*	
C6B	0.8485 (3)	0.3814 (2)	0.17071 (12)	0.0273 (6)	
H6B	0.9439	0.3689	0.1897	0.033*	
C7B	0.7465 (3)	0.30895 (19)	0.17912 (12)	0.0276 (6)	
H7B	0.7723	0.2470	0.2034	0.033*	
C8B	0.6072 (3)	0.32802 (19)	0.15172 (12)	0.0262 (6)	
H8B	0.5371	0.2793	0.1575	0.031*	
C9B	0.5701 (2)	0.41858 (18)	0.11572 (11)	0.0216 (5)	
H9B	0.4745	0.4311	0.0971	0.026*	
C10B	0.6938 (2)	0.87171 (17)	−0.08218 (11)	0.0173 (5)	
C11B	0.7185 (2)	0.86597 (18)	−0.15059 (12)	0.0225 (5)	
F1B	0.78197 (16)	0.78076 (11)	−0.17040 (7)	0.0351 (4)	
C12B	0.6818 (2)	0.9429 (2)	−0.19871 (12)	0.0270 (6)	
H12B	0.6997	0.9364	−0.2452	0.032*	
C13B	0.6180 (2)	1.02988 (19)	−0.17770 (12)	0.0246 (5)	
H13B	0.5918	1.0837	−0.2100	0.029*	
C14B	0.5924 (2)	1.03851 (18)	−0.10960 (12)	0.0226 (5)	
H14B	0.5490	1.0983	−0.0954	0.027*	
C15B	0.6303 (2)	0.95959 (17)	−0.06203 (12)	0.0195 (5)	
H15B	0.6126	0.9660	−0.0155	0.023*	

### Atomic displacement parameters (Å^2^)

**Table:**

	*U*^11^	*U*^22^	*U*^33^	*U*^12^	*U*^13^	*U*^23^
N1A	0.0104 (9)	0.0186 (11)	0.0287 (10)	−0.0008 (7)	0.0050 (8)	0.0038 (8)
C1A	0.0135 (11)	0.0178 (12)	0.0221 (11)	0.0005 (9)	0.0041 (8)	−0.0030 (9)
O1A	0.0112 (8)	0.0213 (9)	0.0336 (9)	−0.0002 (6)	0.0035 (6)	0.0019 (7)
C2A	0.0113 (10)	0.0205 (12)	0.0229 (11)	−0.0017 (9)	0.0041 (8)	−0.0005 (10)
C3A	0.0130 (11)	0.0206 (12)	0.0263 (11)	−0.0028 (9)	0.0038 (8)	−0.0009 (10)
C4A	0.0164 (11)	0.0199 (12)	0.0204 (11)	−0.0013 (9)	0.0014 (8)	−0.0009 (9)
C5A	0.0169 (11)	0.0238 (13)	0.0277 (12)	0.0015 (10)	0.0029 (9)	−0.0015 (11)
C6A	0.0250 (13)	0.0222 (13)	0.0271 (12)	0.0051 (10)	0.0006 (10)	−0.0016 (10)
C7A	0.0327 (14)	0.0178 (13)	0.0222 (12)	−0.0017 (10)	0.0015 (10)	0.0012 (10)
C8A	0.0213 (12)	0.0274 (14)	0.0222 (11)	−0.0066 (10)	0.0046 (9)	0.0001 (10)
C9A	0.0160 (11)	0.0238 (13)	0.0233 (11)	0.0007 (9)	0.0020 (9)	−0.0001 (10)
C10A	0.0092 (10)	0.0185 (12)	0.0273 (12)	0.0032 (8)	0.0072 (8)	0.0016 (10)
C11A	0.0157 (11)	0.0159 (12)	0.0288 (12)	0.0005 (9)	0.0037 (9)	−0.0022 (10)
F1A	0.0282 (8)	0.0204 (8)	0.0366 (8)	−0.0063 (6)	−0.0036 (6)	−0.0016 (6)
C12A	0.0248 (13)	0.0236 (13)	0.0242 (12)	0.0040 (10)	0.0024 (9)	0.0009 (10)
C13A	0.0231 (12)	0.0198 (13)	0.0306 (13)	0.0036 (10)	0.0080 (10)	0.0067 (10)
C14A	0.0171 (11)	0.0167 (12)	0.0327 (13)	0.0005 (9)	0.0094 (9)	−0.0009 (10)
C15A	0.0137 (11)	0.0196 (13)	0.0256 (12)	0.0009 (9)	0.0052 (9)	0.0003 (10)
N1B	0.0106 (9)	0.0186 (11)	0.0275 (10)	0.0030 (8)	0.0052 (8)	0.0026 (8)
C1B	0.0134 (11)	0.0169 (12)	0.0206 (11)	−0.0013 (9)	0.0024 (8)	−0.0041 (9)
O1B	0.0112 (8)	0.0215 (9)	0.0331 (9)	0.0014 (6)	0.0063 (6)	0.0022 (7)
C2B	0.0124 (10)	0.0204 (12)	0.0202 (11)	0.0019 (9)	0.0026 (8)	−0.0021 (9)
C3B	0.0136 (11)	0.0195 (12)	0.0222 (11)	0.0020 (9)	0.0034 (8)	−0.0017 (10)
C4B	0.0165 (11)	0.0216 (13)	0.0178 (11)	0.0021 (9)	0.0062 (8)	−0.0020 (9)
C5B	0.0203 (12)	0.0259 (14)	0.0245 (12)	0.0008 (10)	0.0071 (9)	0.0037 (10)
C6B	0.0230 (13)	0.0332 (15)	0.0264 (12)	0.0073 (11)	0.0064 (10)	0.0067 (11)
C7B	0.0350 (14)	0.0224 (14)	0.0264 (12)	0.0060 (11)	0.0085 (10)	0.0046 (10)
C8B	0.0309 (14)	0.0212 (13)	0.0279 (12)	−0.0049 (10)	0.0089 (10)	−0.0007 (11)
C9B	0.0205 (12)	0.0206 (13)	0.0241 (11)	−0.0006 (9)	0.0046 (9)	−0.0018 (10)
C10B	0.0106 (10)	0.0162 (12)	0.0246 (11)	−0.0028 (8)	0.0010 (8)	0.0018 (9)
C11B	0.0180 (12)	0.0197 (13)	0.0311 (12)	−0.0002 (9)	0.0082 (9)	−0.0026 (10)
F1B	0.0483 (10)	0.0259 (8)	0.0356 (8)	0.0089 (7)	0.0202 (7)	−0.0006 (7)
C12B	0.0280 (13)	0.0281 (14)	0.0254 (12)	−0.0025 (10)	0.0058 (10)	0.0028 (11)
C13B	0.0194 (12)	0.0208 (13)	0.0323 (13)	−0.0034 (9)	0.0009 (9)	0.0092 (11)
C14B	0.0145 (11)	0.0174 (12)	0.0352 (13)	−0.0007 (9)	0.0020 (9)	0.0005 (10)
C15B	0.0137 (11)	0.0191 (12)	0.0259 (11)	−0.0019 (9)	0.0038 (9)	−0.0005 (10)

### Geometric parameters (Å, °)

**Table:**

N1A—C1A	1.360 (3)	N1B—C1B	1.357 (3)
N1A—C10A	1.418 (3)	N1B—C10B	1.414 (3)
N1A—H1NA	0.88 (3)	N1B—H1NB	0.81 (3)
C1A—O1A	1.238 (3)	C1B—O1B	1.237 (3)
C1A—C2A	1.476 (3)	C1B—C2B	1.483 (3)
C2A—C3A	1.335 (3)	C2B—C3B	1.334 (3)
C2A—H2A	0.9500	C2B—H2B	0.9500
C3A—C4A	1.464 (3)	C3B—C4B	1.471 (3)
C3A—H3A	0.9500	C3B—H3B	0.9500
C4A—C5A	1.397 (3)	C4B—C9B	1.397 (3)
C4A—C9A	1.408 (3)	C4B—C5B	1.401 (3)
C5A—C6A	1.394 (3)	C5B—C6B	1.384 (3)
C5A—H5A	0.9500	C5B—H5B	0.9500
C6A—C7A	1.384 (3)	C6B—C7B	1.396 (4)
C6A—H6A	0.9500	C6B—H6B	0.9500
C7A—C8A	1.393 (3)	C7B—C8B	1.388 (3)
C7A—H7A	0.9500	C7B—H7B	0.9500
C8A—C9A	1.383 (3)	C8B—C9B	1.393 (3)
C8A—H8A	0.9500	C8B—H8B	0.9500
C9A—H9A	0.9500	C9B—H9B	0.9500
C10A—C15A	1.389 (3)	C10B—C11B	1.388 (3)
C10A—C11A	1.393 (3)	C10B—C15B	1.389 (3)
C11A—F1A	1.365 (3)	C11B—F1B	1.358 (3)
C11A—C12A	1.373 (3)	C11B—C12B	1.380 (3)
C12A—C13A	1.390 (3)	C12B—C13B	1.386 (4)
C12A—H12A	0.9500	C12B—H12B	0.9500
C13A—C14A	1.390 (3)	C13B—C14B	1.388 (3)
C13A—H13A	0.9500	C13B—H13B	0.9500
C14A—C15A	1.387 (3)	C14B—C15B	1.394 (3)
C14A—H14A	0.9500	C14B—H14B	0.9500
C15A—H15A	0.9500	C15B—H15B	0.9500
			
C1A—N1A—C10A	123.83 (19)	C1B—N1B—C10B	123.75 (19)
C1A—N1A—H1NA	119.1 (16)	C1B—N1B—H1NB	119.8 (19)
C10A—N1A—H1NA	117.1 (16)	C10B—N1B—H1NB	116.4 (19)
O1A—C1A—N1A	122.1 (2)	O1B—C1B—N1B	122.1 (2)
O1A—C1A—C2A	123.66 (19)	O1B—C1B—C2B	123.6 (2)
N1A—C1A—C2A	114.22 (19)	N1B—C1B—C2B	114.22 (19)
C3A—C2A—C1A	120.7 (2)	C3B—C2B—C1B	120.8 (2)
C3A—C2A—H2A	119.6	C3B—C2B—H2B	119.6
C1A—C2A—H2A	119.6	C1B—C2B—H2B	119.6
C2A—C3A—C4A	128.3 (2)	C2B—C3B—C4B	127.4 (2)
C2A—C3A—H3A	115.9	C2B—C3B—H3B	116.3
C4A—C3A—H3A	115.9	C4B—C3B—H3B	116.3
C5A—C4A—C9A	118.1 (2)	C9B—C4B—C5B	118.4 (2)
C5A—C4A—C3A	118.9 (2)	C9B—C4B—C3B	119.18 (19)
C9A—C4A—C3A	123.0 (2)	C5B—C4B—C3B	122.5 (2)
C6A—C5A—C4A	120.9 (2)	C6B—C5B—C4B	120.8 (2)
C6A—C5A—H5A	119.6	C6B—C5B—H5B	119.6
C4A—C5A—H5A	119.6	C4B—C5B—H5B	119.6
C7A—C6A—C5A	120.3 (2)	C5B—C6B—C7B	120.4 (2)
C7A—C6A—H6A	119.8	C5B—C6B—H6B	119.8
C5A—C6A—H6A	119.8	C7B—C6B—H6B	119.8
C6A—C7A—C8A	119.5 (2)	C8B—C7B—C6B	119.5 (2)
C6A—C7A—H7A	120.2	C8B—C7B—H7B	120.2
C8A—C7A—H7A	120.2	C6B—C7B—H7B	120.2
C9A—C8A—C7A	120.4 (2)	C7B—C8B—C9B	120.0 (2)
C9A—C8A—H8A	119.8	C7B—C8B—H8B	120.0
C7A—C8A—H8A	119.8	C9B—C8B—H8B	120.0
C8A—C9A—C4A	120.8 (2)	C8B—C9B—C4B	120.9 (2)
C8A—C9A—H9A	119.6	C8B—C9B—H9B	119.5
C4A—C9A—H9A	119.6	C4B—C9B—H9B	119.5
C15A—C10A—C11A	117.6 (2)	C11B—C10B—C15B	117.8 (2)
C15A—C10A—N1A	122.8 (2)	C11B—C10B—N1B	119.8 (2)
C11A—C10A—N1A	119.7 (2)	C15B—C10B—N1B	122.4 (2)
F1A—C11A—C12A	118.89 (19)	F1B—C11B—C12B	119.1 (2)
F1A—C11A—C10A	118.1 (2)	F1B—C11B—C10B	118.1 (2)
C12A—C11A—C10A	123.0 (2)	C12B—C11B—C10B	122.8 (2)
C11A—C12A—C13A	118.5 (2)	C11B—C12B—C13B	118.6 (2)
C11A—C12A—H12A	120.7	C11B—C12B—H12B	120.7
C13A—C12A—H12A	120.7	C13B—C12B—H12B	120.7
C14A—C13A—C12A	120.0 (2)	C12B—C13B—C14B	120.2 (2)
C14A—C13A—H13A	120.0	C12B—C13B—H13B	119.9
C12A—C13A—H13A	120.0	C14B—C13B—H13B	119.9
C15A—C14A—C13A	120.3 (2)	C13B—C14B—C15B	120.1 (2)
C15A—C14A—H14A	119.8	C13B—C14B—H14B	119.9
C13A—C14A—H14A	119.8	C15B—C14B—H14B	119.9
C14A—C15A—C10A	120.6 (2)	C10B—C15B—C14B	120.5 (2)
C14A—C15A—H15A	119.7	C10B—C15B—H15B	119.7
C10A—C15A—H15A	119.7	C14B—C15B—H15B	119.7

### Hydrogen-bond geometry (Å, °)

**Table:**

CgA and CgB are the centroids of the fluorobenzene rings in molecules A and B respectively.

**Table:**

*D*—H···*A*	*D*—H	H···*A*	*D*···*A*	*D*—H···*A*
N1A—H1NA···O1B	0.88 (3)	1.98 (3)	2.851 (2)	173 (2)
N1B—H1NB···O1A^i^	0.81 (3)	2.07 (3)	2.870 (2)	170 (2)
C14A—H14A···O1A^ii^	0.95	2.50	3.410 (3)	160
C14B—H14B···O1B^iii^	0.95	2.59	3.476 (3)	155
C9B—H9B···CgA^iv^	0.95	2.89	3.679 (2)	141
C5A—H5A···CgB^v^	0.95	2.80	3.621 (2)	149

Symmetry codes: (i) *x*+1, *y*, *z*; (ii) −*x*, −*y*+1, −*z*; (iii) −*x*+1, −*y*+2, −*z*; (iv) *x*, −*y*+1/2, *z*+1/2; (v) *x*+1, −*y*+1/2, *z*−1/2.
